# EP9158H: An Immunoinformatics-Designed mRNA Vaccine Encoding Multi-Epitope Antigens and Dual TLR Agonists for Tuberculosis Prevention

**DOI:** 10.3390/bioengineering12121378

**Published:** 2025-12-18

**Authors:** Mingming Zhang, Syed Luqman Ali, Yuan Tian, Aigul Abduldayeva, Shuang Zhou, Yajing An, Yufeng Li, Ruizi Ni, Lingxia Zhang, Yanhua Liu, Weiguo Sun, Wenping Gong

**Affiliations:** 1 Senior Department of Tuberculosis, Chinese PLA General Hospital, Beijing 100091, China; zhangmingming@stu.sqxy.edu.cn (M.Z.); zhoushuang@stu.sqxy.edu.cn (S.Z.); anyajing@asu.edu.pl (Y.A.); liyufe@stu.sqxy.edu.cn (Y.L.); ruizini@asu.edu.pl (R.N.); 1707025046@stu.sqxy.edu.cn (L.Z.); 1707025045@stu.sqxy.edu.cn (Y.L.); 2 Graduate School, Hebei North University, Zhangjiakou 075000, China; tianyuan@stu.sqxy.edu.cn; 3 Department of Biochemistry, Abdul Wali Khan University, Mardan 23200, Pakistan; luqmanali@awkum.edu.pk; 4 Department of Research Institute of Preventive Medicine Named Academician E. Dalenov, Astana Medical University, Astana 010000, Kazakhstan; abduldayeva.a@amu.kz; 5 Department of Geriatrics, The Eighth Medical Center of PLA General Hospital, Beijing 100091, China

**Keywords:** tuberculosis, mRNA vaccine, immunoinformatics, TLR agonist, molecular dynamics, immune simulation

## Abstract

**Background**: Tuberculosis (TB) remains a pressing global health crisis. The inadequate efficacy of the BCG vaccine against adult pulmonary TB underscores the urgent need for novel, effective vaccines. This study aimed to design a novel mRNA vaccine candidate against TB using a rational immunoinformatics approach. **Methods**: From 13 antigens, >12,000 epitopes were filtered to select 60 optimal peptides (36 CTL, 16 HTL, 8 B-cell), assembled into 25 scaffolds with 49 TLR2/4 agonist configurations. EP9158H underwent structural modeling, 100 ns molecular dynamics, docking, immune simulation, RNAfold, and conservation analysis across 76 strains. **Results**: EP9158H, encoding 15 CTL, 9 HTL, and 8 B-cell epitopes flanked by TLR2 agonist ESAT-6 and TLR4 agonist HBHA, emerged as the optimal candidate. All 32 constituent epitopes showed >81% conservation, with 81.25% exhibiting perfect identity across MTBC lineages. The scaffold demonstrated high solubility (0.531), broad population coverage (73.76% MHC-I, 88.91% MHC-II), optimal TLR2/4 docking scores (−1359.7 and −1348.3), and robust structural stability (ProSA Z-score −6.18; RMSD 22–27 Å). Immune simulation predicted strong Th1-biased T-cell responses and high levels of antibody titers. RNAfold analysis revealed stable mRNA secondary structures (MFE −1127.5 kcal/mol) supporting efficient translation. **Conclusions**: EP9158H integrates broad epitope coverage, dual TLR agonism, and validated stability. Compared to single-antigen vaccines, it offers superior strain coverage, enhanced innate activation, and mRNA advantages for CTL induction, warranting experimental validation.

## 1. Introduction

Tuberculosis (TB), caused by *Mycobacterium tuberculosis* (MTB), remains a protracted global health emergency [1,2]. Despite being both preventable and curable, TB claimed 1.25 million lives in 2023 and contributed to 10.8 million incident cases, outranking any other single infectious agent apart from SARS-CoV-2 [2]. The only licensed prophylactic, live-attenuated *Mycobacterium bovis* Bacille Calmette–Guérin (BCG), confers consistent protection against disseminated childhood TB but exhibits highly variable—and often negligible—efficacy against pulmonary disease in adults, the main transmission source [3,4,5]. Consequently, BCG roll-out has failed to interrupt community circulation of MTB, and low-efficacy cohorts act as persistent reservoirs that fuel the pandemic. Achieving the WHO “End TB” milestones (≥90% incidence and ≥95% mortality reduction by 2035) therefore demands next-generation vaccines that reliably block adult pulmonary disease and curtail transmission [6].

Over the past two decades, more than 20 vaccine constructs—ranging from recombinant BCG overexpressing immunodominant antigens to subunit protein-adjuvant formulations and viral vectors—have entered clinical evaluation [7,8,9,10]. Disappointingly, only the M72/AS01E candidate recently demonstrated 49.7% protective efficacy in a Phase IIb proof-of-concept trial, and no regimen has yet progressed to licensure [11]. Repeated failures underscore three translational bottlenecks: (i) an incomplete antigenic repertoire that omits latent-phase or strain-variable targets, (ii) suboptimal engagement of CD8^+^ cytotoxic T lymphocytes (CTL) required for intracellular bacterial clearance, and (iii) inadequate innate sensing that limits the magnitude and durability of adaptive immunity. Novel platforms that integrate in silico selection of immunodominant epitopes with intrinsic adjuvanticity are therefore urgently needed to break this attrition cycle [12,13].

mRNA vaccines have emerged as an agile modality capable of addressing these exact gaps [14,15,16]. The development and production cycle of mRNA vaccines is extremely short. Once the gene sequence encoding the target antigen is obtained, mRNA can be synthesized in vitro through transcription. The technology for in vitro transcription of mRNA vaccines is already well-established. If a viral strain mutates, only the mRNA sequence needs to be updated—there is no need to redesign the entire production process. Compared with protein-based vaccines and viral-vector vaccines, mRNA vaccines can be produced far more rapidly and conveniently, highlighting their flexibility and efficiency. By delivering lipid-encapsulated nucleoside-modified transcripts directly into host cytosol, mRNA directs the synthesis of the target antigenic protein exclusively in the cytoplasm; it does not enter the nucleus and is not integrated into the host’s genomic DNA, conferring a very high level of biological safety [17]. mRNA vaccines enable endogenous antigen synthesis, major histocompatibility complex (MHC) class I presentation, and robust CTL induction without the biosafety constraints of live vectors [18,19,20]. Sequence plasticity allows multiplexed expression of several antigens or epitope strings, while rapid, cell-free manufacturing facilitates iterative updates that track MTB evolution [21,22]. The first proof-of-concept was reported in 2004, when Tascon and colleagues demonstrated that four intramuscular injections of nude in vitro-transcribed (IVT) mRNA encoding the immunodominant antigen MPT83 elicited measurable protection against MTB challenge in mice [23]. Six years later, the São Paulo group extended this observation to the nasal route, showing that a single 10 µg dose of mRNA-Hsp65 reduced pulmonary bacterial loads and inflammation in a murine TB model [24]. More recently, Larsen SE et al. combined a self-amplifying replicon RNA (repRNA) encoding the multivalent ID91 antigen with a first-generation nanostructured lipid carrier and documented modest but reproducible immunogenicity and protection in mice [25]. Parallel computational efforts have further expanded the toolkit, demonstrating that immunoinformatics-driven epitope mapping, multi-antigen string design, and in silico immune simulation can accelerate candidate selection and optimize population coverage [22,26,27,28,29,30,31,32,33,34,35,36]. Translation to the clinic is now underway: BioNTech has opened two phase I trials (NCT05537038; NCT05547464) evaluating lipid-nanoparticle-formulated mRNA candidates, although neither has disclosed efficacy nor detailed immunological read-outs. Collectively, these studies establish that mRNA vaccines can engage the immune machinery required to control MTB; however, a coherent, antigen-agnostic design platform that integrates epitope breadth, built-in adjuvanticity, and population-wide HLA promiscuity remains conspicuously absent.

Here, we apply an integrative immunoinformatics workflow to design a novel mRNA vaccine candidate against MTB. By combining epitope prediction, structural modeling, and immune simulation, we construct a multivalent mRNA construct aimed at eliciting robust and broad-spectrum immune responses. This study offers a rational, modular framework for TB vaccine design and highlights the potential of mRNA technology in addressing persistent challenges in tuberculosis prevention.

## 2. Materials and Methods

### 2.1. Antigen Selection and In Silico Quality Control

Thirteen MTB antigens (Rv0287, Rv0288, Rv1009, Rv1733c, Rv1886c, Rv2006, Rv2450c, Rv3131, Rv3619c, Rv3803c, Rv3804c, Rv3873, Rv3876) were retrieved from UniProt (https://www.uniprot.org, release 2023_03, accessed on 10 February 2025). Antigenicity was evaluated with VaxiJen v2.0 (http://www.ddg-pharmfac.net/vaxijen/VaxiJen/VaxiJen.html, threshold 0.4, antigens with a value greater than 0.4 are considered to have relatively high antigenicity, accessed on 12 February 2025) [37], allergenicity with AllerTop v1.1 (https://ddg-pharmfac.net/allertop/, accessed on 12 February 2025) and toxicity with ToxinPred2 (https://webs.iiitd.edu.in/raghava/toxinpred2/batch.html, accessed on 12 February 2025) [38]; sequences meeting antigenicity ≥ 0.4, non-toxic, and non-allergenic criteria were retained.

### 2.2. Subcellular Localization, Signal Peptide, and Trans-Membrane Topology

SignalP-6.0 (https://services.healthtech.dtu.dk/services/SignalP-6.0/, version 6.0e, accessed on 13 February 2025), TOPCONS (https://topcons.net/pred/, v2.1, accessed on 13 February 2025), and DeepTMHMM (https://dtu.biolib.com/DeepTMHMM/, v1.0.25, accessed on 13 February 2025) were used for signal-peptide and TM-helix prediction. Subcellular localization was assigned with DeepLocPro (https://services.healthtech.dtu.dk/services/DeepLocPro-1.0/, v1.0, accessed on 13 February 2025) [39] and TBpred (https://webs.iiitd.edu.in/raghava/tbpred/, v1.0, accessed on 13 February 2025).

### 2.3. T-Cell and Linear B-Cell Epitope Prediction

Cytotoxic T-lymphocyte (CTL) candidates were identified among 9- and 10-mer peptides using the IEDB MHC-I prediction portal (http://tools.iedb.org/mhci/, accessed on 15 February 2025) with NetMHCpan 4.1 EL and BA modes; peptides were retained only when the EL percentile rank was below 0.5, the BA IC_50_ value was under 50 nM and the IEDB immunogenicity score exceeded 0 [40,41]. The NetMHCpan 4.1 EL model outputs a percentile rank; the smaller the rank, the higher the predicted affinity. The BA model gives IC_50_ values in nM; peptides with IC_50_ < 50 nM are regarded as high-affinity binders. Helper T-lymphocyte (HTL) epitopes of 12–18 residues were scanned with the IEDB MHC-II server (http://tools.iedb.org/mhcii/, accessed on 15 February 2025) using NetMHCIIpan 4.1 EL with the same 0.5 percentile cut-off [40]. The NetMHCIIpan 4.1 EL model likewise returns a percentile rank, where a lower rank indicates higher predicted affinity. The cytokine signature of each HTL peptide was then examined with IFN-Epitope (https://webs.iiitd.edu.in/raghava/ifnepitope/predict.php, accessed on 27 February 2025), IL4pred (https://webs.iiitd.edu.in/raghava/il4pred/predict.php, accessed on 27 February 2025), and IL10pred (https://webs.iiitd.edu.in/raghava/il10pred/predict3.php, accessed on 27 February 2025); only sequences predicted as IFN-γ positive, IL-4 negative, and IL-10 negative were carried forward. Every T-cell epitope was subsequently rescreened for antigenicity (VaxiJen v2.0, threshold ≥ 0.5 [37]), toxicity (ToxinPred3, https://webs.iiitd.edu.in/raghava/toxinpred3/, accessed on 27 February 2025), and allergenicity (AllerTop v1.1 and v2.1, http://www.ddg-pharmfac.net/AllerTOP/, accessed on 27 February 2025). Linear B-cell epitopes were predicted as 16-mers with ABCpred (https://webs.iiitd.edu.in/raghava/abcpred/ABC_submission.html, v1.0, accessed on 27 February 2025) using the default threshold of 0.51 [42]. The server indicates that a threshold greater than 0.5 provides good sensitivity and specificity. The highest-scoring peptide for each antigen was retained after identical safety filtering.

To assess the potential cross-strain coverage of the designed vaccine, the amino acid sequences of all constituent CTL, HTL, and B-cell epitopes were individually analyzed for homology against a broad panel of Mycobacterium strains. The protein BLAST (BLASTP) algorithm was employed against the non-redundant protein sequence (nr) database, restricted to the genus *Mycobacterium* (taxid: 1763), using the NCBI online platform. The analysis focused on identifying the percentage of sequence identity for each epitope across major lineages of the *Mycobacterium tuberculosis* complex (MTBC) and other clinically relevant non-tuberculous mycobacteria.

### 2.4. Multi-Epitope String Assembly and Scaffold Generation

Selected epitopes were concatenated with linkers AAY (CTL), GPGPG (HTL), and KK (B-cell). PADRE peptide (AGLFQRHGEGTKATVGEPV) and a C-terminal 6 × His tag were appended; an N-terminal TLR agonist (RpIL) was inserted via EAAAK. Twenty-five scaffold variants (640–710 aa) were built and screened for solubility (Protein-Sol, https://protein-sol.manchester.ac.uk/, threshold ≥ 0.45, accessed on 6 March 2025) [43], Class-I immunogenicity (http://tools.iedb.org/immunogenicity/, accessed on 10 March 2025), antigenicity (VaxiJen), toxicity (ToxinPred2), allergenicity (AllerTop), and global HLA coverage (http://tools.iedb.org/population/, accessed on 10 March 2025). Physicochemical parameters were computed with ProtParam (https://web.expasy.org/protparam/, v1.2, accessed on 10 March 2025).

### 2.5. TLR-Agonist Library Construction and Down-Selection

Having fixed the best multi-epitope scaffold, we next created a combinatorial library in which TLR agonists were genetically fused to the N- or C-terminus to provide built-in adjuvanticity. Four TLR-2 agonists—ESAT-6, PSMα4, Pam2Cys, and PorB—and five TLR-4 agonists—RpfE, RpIL, HBHA, CTB, and RS-09—were individually appended to the best multi-epitope scaffold together with the PADRE helper peptide, yielding nine mono-adjuvanted candidates. To examine synergistic effects, we then generated twenty dual-adjuvanted constructs by pairing one TLR-2 and one TLR-4 agonist in a single polypeptide; each pair was assembled in both N→C and C→N orientations, yielding an additional twenty sequences. The complete library, therefore, comprised forty-nine distinct sequences, all carrying an N-terminal EAAAK linker, an internal PADRE sequence, and a C-terminal 6 × His tag. Every member was submitted to an identical in silico quality pipeline: antigenicity (VaxiJen v2.0, threshold 0.4), Class-I immunogenicity (IEDB), solubility (Protein-Sol, threshold 0.45), toxicity (ToxinPred2), and allergenicity (AllerTop v1.1 and v2.1) were evaluated, and physicochemical descriptors, including molecular weight, theoretical pI, aliphatic index, GRAVY, and instability index, were extracted with ProtParam. Constructs that passed every filter were advanced to structure prediction and docking against TLR-2 and TLR-4 to identify the optimal adjuvant configuration for subsequent experimental work.

### 2.6. Structure Prediction, Refinement, and Validation

Secondary-structure profiles were generated with PSIPRED (http://bioinf.cs.ucl.ac.uk/psipred/, v4.0, accessed on 17 March 2025) and SOPMA (https://npsa.lyon.inserm.fr/cgi-bin/npsa_automat.pl?page=/NPSA/npsa_sopma.html, accessed on 17 March 2025) using their default window lengths and similarity cut-offs. For tertiary modeling, AlphaFold-3 (https://golgi.sandbox.google.com/, AF3-2024.1, accessed on 17 March 2025) was employed with the full multiple-sequence alignment pipeline; the resulting models were subjected to five cycles of side-chain repacking and backbone relaxation in GalaxyRefine (https://galaxy.seoklab.org/cgi-bin/submit.cgi?type=REFINE, v4.0, accessed on 17 March 2025). Global model quality was assessed via the ProSA-web server (https://prosa.services.came.sbg.ac.at/prosa.php, accessed on 20 March 2025), recording the standard Z-score, while local geometry was inspected with the Ramachandran module of UCLA-DOE SAVES v6.0 (https://saves.mbi.ucla.edu/, accessed on 20 March 2025). Refinement comparisons and final images were prepared in ChimeraX 1.9.

### 2.7. Molecular-Dynamics Simulation Protocol

To assess the stability of the vaccine–TLR complexes, we performed 100 ns MD using the Desmond module (Schrödinger 2023-3) with the OPLS4 force field. The system was prepared in Maestro 13.6 using the Protein Preparation Wizard, immersed in an orthorhombic SPC-water box extending 10 Å beyond any protein atom, and neutralized with 0.15 M NaCl. After default restrained minimization and the four-step Desmond relaxation protocol, production was run in the NPT ensemble at 300 K and 1 atm with a 2-fs time-step; bonds to hydrogen were constrained with M-SHAKE. Long-range electrostatics were handled by particle-mesh Ewald (9 Å cut-off), and temperature and pressure were maintained with the Nosé-Hoover thermostat and Martyna–Tobias–Klein barostat. Trajectories were saved every 100 ps and converted to XTC with MDTraj 1.9.9 for subsequent analysis.

### 2.8. Post-MD Trajectory Analyses

Cα RMSD, RMSF, and radius of gyration were computed with GROMACS 2023.3 after least-squares superposition. For principal component analysis, the covariance matrix was built on Cα atoms with gmx covar and diagonalized with gmx anaeig; the trajectory was then projected onto the first two eigenvectors. A two-dimensional free-energy landscape along PC1 and PC2 was constructed with gmx sham (bin width 0.1 nm) and smoothed by LOESS in R. Dynamic cross-correlation matrices were generated with Bio3D 2.4.0 from the DCD trajectory exported by MDTraj.

### 2.9. Discontinuous B-Cell Epitope Prediction

The refined AlphaFold-3 structure of EP9158H was analyzed with ElliPro (http://tools.iedb.org/ellipro/, v2.0, accessed on 31 March 2025) using the default residue-contact threshold of 0.693 Å; predicted discontinuous epitopes were ranked by protrusion index.

### 2.10. In Silico Immune Simulation

Immune responses were simulated with C-ImmSim (https://150.146.2.1/C-IMMSIM/index.php, v10.1, accessed on 2 July 2025) using a PSSM approach. Parameters were set to simulation volume 50, 1050-time steps (1 step = 8 h), injections at steps 1, 90, and 180, and random seed 42; all remaining settings were left at server defaults. Output included cell counts, cytokine levels, and antibody titers over 300 simulated days.

### 2.11. Codon Optimization, mRNA Folding, and mRNA Structure Construction

The gene was optimized for Homo sapiens with ExpOptimizer (https://www.novoprolabs.com/tools/codon-optimization, accessed on 16 September 2025), reporting CAI and GC %. The RNA sequence was generated with DNA→RNA→Protein (https://biomodel.uah.es/en/lab/cybertory/analysis/trans.htm, accessed on 16 September 2025) and folded with RNAfold (http://rna.tbi.univie.ac.at/cgi-bin/RNAWebSuite/RNAfold.cgi, v2.6.4, accessed on 16 September 2025), yielding MFE, centroid, and ensemble free energies. To ensure proper translation after vaccination, we engineer the EP9158H mRNA with an m^7^G cap at the 5′ end and a poly-A tail at the 3′ end for structural stability, and flank the coding sequence with a 5′ UTR, Kozak sequence, TPA signal peptide, MITD sequence, and a 3′ UTR to guarantee efficient expression of the target protein.

## 3. Results

### 3.1. From 13 Clinical Antigens to Eight Candidates: A Three-Step Toxicity–Localization–Accessibility Filter

**Figure 1:**
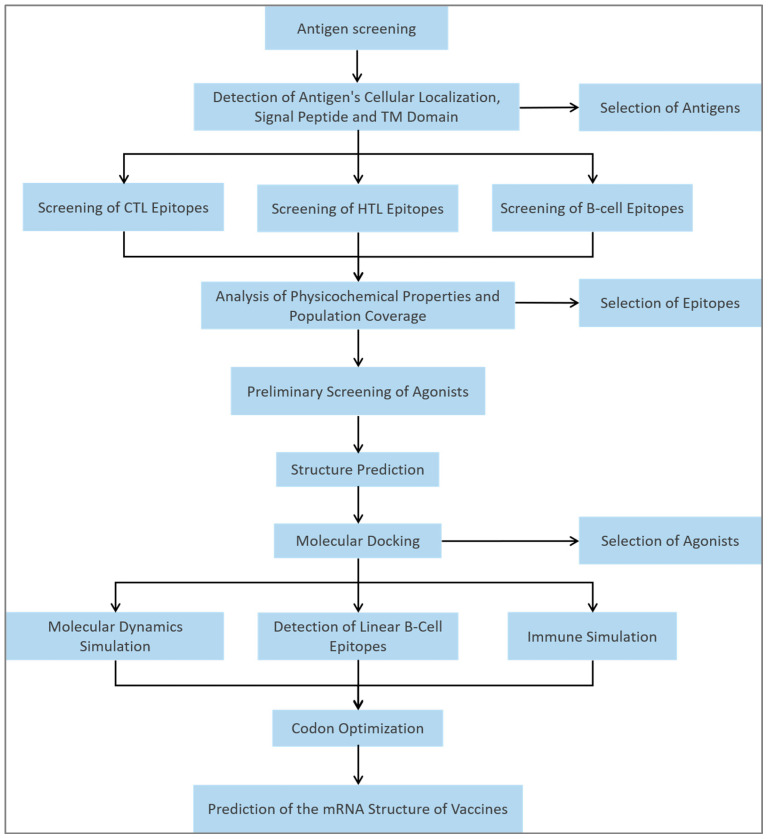
Workflow for the immunoinformatics-driven design of the EP9158H mRNA vaccine. The schematic illustrates the multi-step computational pipeline, from antigen selection and epitope prediction to final vaccine construct assembly and evaluation.

The starting panel comprised thirteen MTB antigens that have entered pre-clinical or clinical evaluation: Rv0287, Rv0288, Rv1009, Rv1733c, Rv1886c, Rv2006, Rv2450c, Rv3131, Rv3619c, Rv3803c, Rv3804c, Rv3873, and Rv3876. First, a safety-and-exposure double gate was applied: VaxiJen > 0.4 plus simultaneous non-toxic and non-allergenic calls by ToxinPred and AllerTop; all thirteen antigens passed (Table 1). We next focused on accessibility—only antigens that the host immune system can encounter first were considered valuable. Combining SignalP 6.0, TOPCONS, and DeepTMHMM, seven antigens carried signal peptides or transmembrane domains (Rv1009, Rv1733c, Rv1886c, Rv2450c, Rv3803c, Rv3804c, Rv3873); DeepLocPro and TBpred further classified five as secreted and two as membrane-anchored. To cover the intracellular replication phase, the highly expressed cytosolic Rv2006 was added. Ultimately, eight antigens (seven secreted/membrane-anchored and one cytosolic high-expressor) advanced to epitope mining; the flow chart is shown in Figure 1.

### 3.2. Parallel Screening and Numerical Contraction of CTL, HTL, and Linear B-Cell Epitopes

Epitopes were predicted for the eight selected antigens with NetMHCpan 4.1 (CTL), NetMHCIIpan 4.1 (HTL), and ABCpred (B-cell), applying identical two-tier filtering: tier 1 set affinity/immunogenicity thresholds; tier 2 removed toxic, allergenic, or incorrectly cytokine-polarized peptides. Details by epitope type are given below.

#### 3.2.1. CTL Epitopes

**Figure 2:**
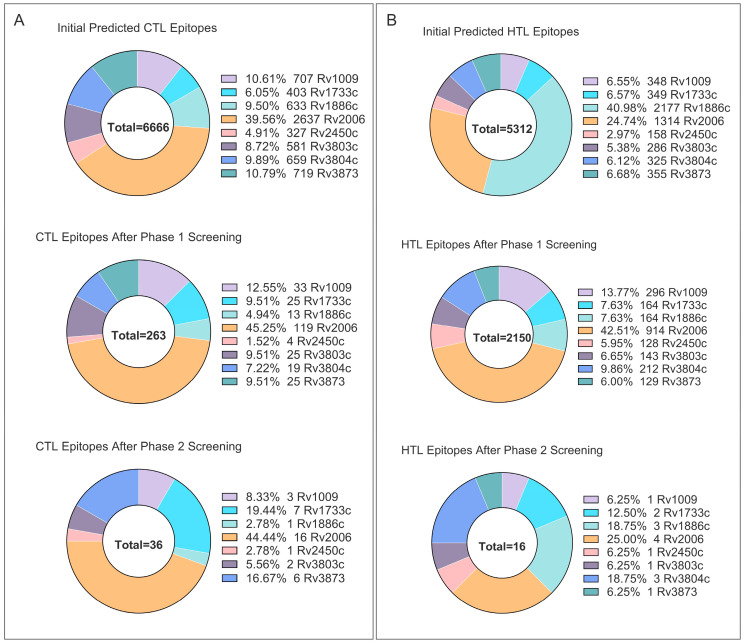
In silico prediction and filtering of T-cell epitopes. (**A**) Number of predicted cytotoxic T-lymphocyte (CTL) epitopes for each of the eight selected antigens before and after applying affinity, immunogenicity, and safety filters. (**B**) Number of predicted helper T-lymphocyte (HTL) epitopes before and after filtering based on MHC-II binding affinity, cytokine induction profile (IFN-γ^+^/IL-4^−^/IL-10^−^), and safety.

NetMHCpan 4.1 EL and BA jointly returned 6666 9–10 mer peptides (Rv1009 707, Rv1733c 403, Rv1886c 633, Rv2006 2637, Rv2450c 327, Rv3803c 581, Rv3804c 659, Rv3873 719; Figure 2A). Tier 1 kept peptides with EL percentile < 0.5%, BA IC_50_ < 50 nM, and IEDB immunogenicity score > 0, leaving 263 peptides (33, 25, 13, 119, 4, 25, 19, 25 per antigen). Tier 2 added VaxiJen > 0.5, ToxinPred non-toxic and AllerTop non-allergenic filters, yielding a final set of 36 CTL epitopes (3, 7, 1, 16, 1, 2, 0, 6); sequences and parameters are listed in Table S1.

#### 3.2.2. HTL Epitopes

NetMHCIIpan 4.1 EL (12–18 mer) produced 5312 peptides (Rv1009 348, Rv1733c 349, Rv1886c 2177, Rv2006 1314, Rv2450c 158, Rv3803c 286, Rv3804c 325, Rv3873 355; Figure 2B). Tier 1 (EL percentile < 0.5%) retained 2150 peptides (296, 164, 164, 914, 128, 143, 212, 129). Tier 2 required IFN-γ prediction positive, IL-4 and IL-10 negative, plus VaxiJen > 0.5, non-toxic and non-allergenic, giving 16 HTL epitopes (1, 2, 3, 4, 1, 1, 3, 1); sequences and parameters are given in Table S2.

#### 3.2.3. Linear B-Cell Epitopes

ABCpred (16 mer, threshold 0.51) predicted 353 peptides (Rv1009 39, Rv1733c 21, Rv1886c 38, Rv2006 125, Rv2450c 19, Rv3803c 34, Rv3804c 38, Rv3873 39). After toxicity and allergenicity filtering, 117 peptides remained (Table S3). To limit vaccine length, only the top-scoring peptide per antigen that passed safety checks was kept, yielding 8 B-cell epitopes for final assembly.

In summary, the above pipeline reduced >12,000 primary predictions to 36 CTL, 16 HTL, and 8 B-cell epitopes—60 peptides in total—for construction of the vaccine scaffold.

#### 3.2.4. Conservation Analysis of Vaccine Epitopes Across Mycobacterium Strains

**Figure 3:**
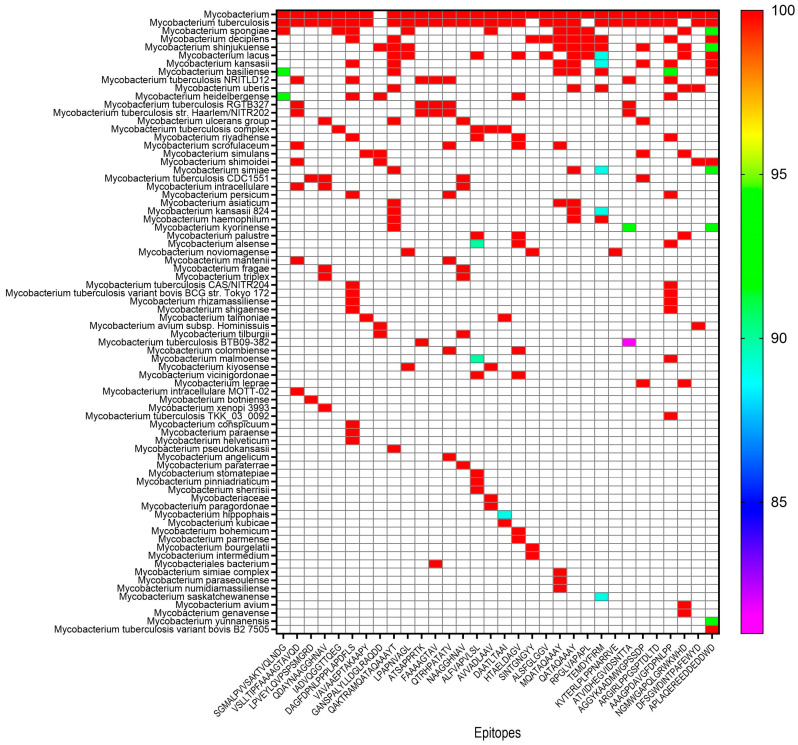
Conservation landscape of the EP9158H vaccine epitopes across Mycobacterium strains. Heatmap showing the percentage of sequence identity for each of the 32 vaccine epitopes (*x*-axis) against 76 Mycobacterium strains (*y*-axis). Epitopes are grouped by type (HTL, CTL, B-cell). Identity values are color-coded from red (high identity) to pink (low identity), based on pairwise BLASTP analysis. White cells indicate that no significant homology was detected for the epitope in the corresponding strain.

To evaluate the potential for the EP9158H vaccine to confer broad-spectrum immunity, we analyzed the sequence conservation of its 32 constituent epitopes across 76 diverse Mycobacterium strains. The conservation analysis yielded a highly favorable result. The vast majority of the vaccine’s epitopes demonstrated perfect sequence conservation. Specifically, 26 out of the 32 epitopes (81.25%) exhibited 100% sequence identity across all 76 mycobacterial strains tested (Figure 3). This indicates that these core components of the vaccine are invariant across a wide phylogenetic range of pathogens, including major lineages of the MTBC and numerous non-tuberculous mycobacteria. Among the remaining six epitopes, sequence identity was still high, ranging from 81% to 100%. Only one epitope, the B-cell epitope ATVIDHEGVIDSNTTA, showed a sequence identity below 90%, with a minimum of 81% in Mycobacterium tuberculosis BTB09-382. The other five epitopes all maintained identity levels of 89% or higher in the strains, where they were not fully conserved.

### 3.3. Scoring and Selection of the Vaccine Scaffold: mRV12 Tops Four Quantitative Indices

To determine the best epitope order, the 60 shortlisted peptides (36 CTL, 16 HTL, 8 B) were shuffled into 25 multi-epitope frameworks (termed mRV1–mRV25, Table S4). Every construct spanned 640–710 amino acids, started with an EAAAK-RpIL leader and ended with a 6 × His tag; linkers were fixed as AAY between CTL, GPGPG between HTL, and KK between B-cell epitopes. Dissolution capacity, Class I immunogenicity, instability index, and worldwide HLA coverage were set as the first-round hard filters; numerical values are collected in Table S4.

**Figure 4:**
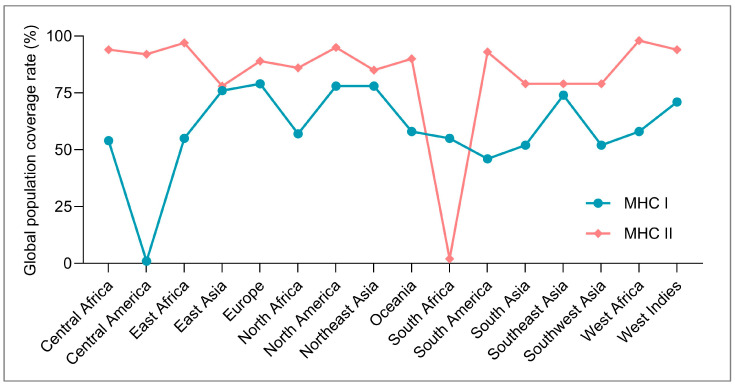
Global population coverage of the selected epitope repertoire. The bar graph shows the projected coverage of the combined MHC Class I and II epitopes across different geographic regions, demonstrating the vaccine’s broad potential applicability.

mRV12 satisfied all cut-offs and delivered the highest composite score: (i) Protein-Sol solubility 0.506 (threshold ≥ 0.45); (ii) IEDB Class I immunogenicity 4.939 (threshold ≥ 4.0); (iii) ProtParam instability index 32.8 (<40 is stable); and (iv) population coverage 73.76% (Class I) and 88.91% (Class II)—the top value among the 25 frameworks (Figure 4). Additional physicochemical indices—MW 69.30 kDa, pI 5.31, GRAVY −0.159, and a 30h half-life in mammalian reticulocytes—fall within the ranges favorable for soluble expression and efficient presentation. Consequently, the epitope composition of mRV12 (15 CTL, 9 HTL, 8 B) was chosen as the universal backbone for downstream agonist grafting (Table 2).

### 3.4. Agonist Knock-Out Derby: One Optimal Solution Out of 49 Designs (EP9158H)

Starting from the mRV12 multi-epitope scaffold, we systematically grafted Toll-like receptor agonists to either the N- or C-terminus, yielding 49 candidate vaccine sequences (Table S5). The design was carried out in two stages: first, four TLR-2 agonists (ESAT-6, PSMα4, Pam_2_Cys, and PorB) and five TLR-4 agonists (RpfE, RpIL, HBHA, CTB, and RS-09) were fused individually to create nine mono-agonist constructs; second, every possible TLR-2/TLR-4 pair was introduced simultaneously, generating twenty fixed-order dual-agonist sequences, after which the N/C positions of each pair were swapped to produce a further twenty mirrored combinations, giving a total of forty dual-agonist candidates. All constructs retained the PADRE helper peptide (AGLFQRHGEGTKATVGEPV) and a C-terminal 6 × His tag to ensure uniform purification and adjuvanticity.

**Figure 5:**
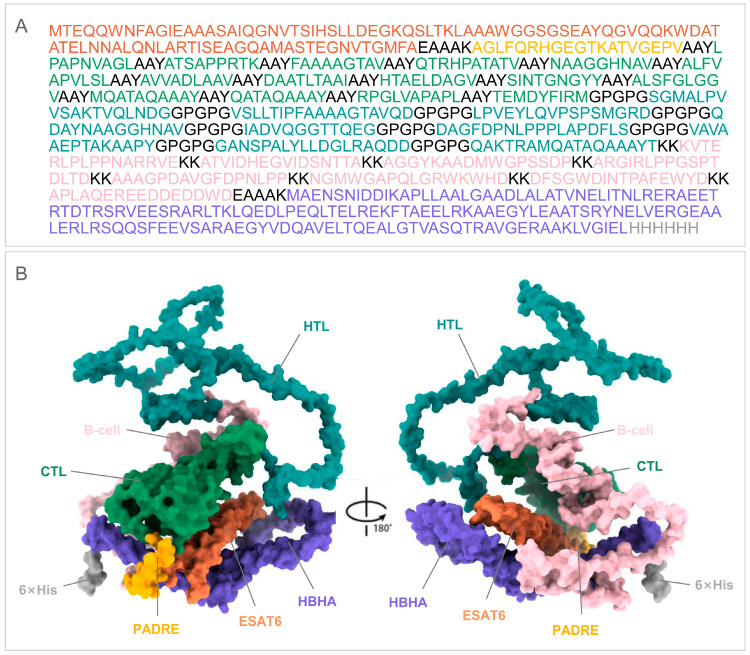
Primary sequence and predicted 3D structure of the EP9158H vaccine protein. (**A**) Linear amino acid sequence of EP9158H, color-coded by component (e.g., linkers, epitopes, adjuvants). (**B**) The tertiary structure of EP9158H predicted by AlphaFold3 and visualized using ChimeraX.

**Figure 6:**
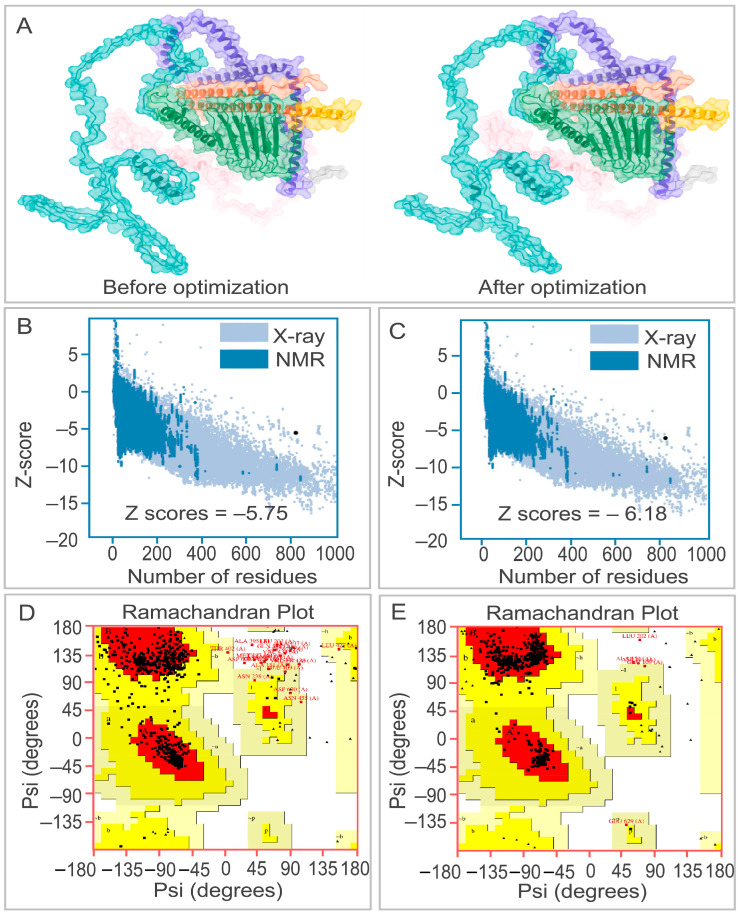
Structural refinement and validation of the EP9158H model. (**A**) Superposition of the vaccine structure before (left) and after (right) refinement with GalaxyRefine, showing improved side-chain packing and backbone stability. (**B**,**C**) ProSA-web Z-scores for the model before (−5.75) and after (−6.18) refinement, indicating overall model quality within the range of native proteins. (**D**,**E**) Ramachandran plots for the refined model, showing the distribution of phi and psi angles with residues in most favored regions (dark red), additionally allowed regions (yellow), and disallowed regions (white).

A first-round physicochemical screen applied five non-negotiable filters: Protein-Sol solubility > 0.45, IEDB Class I immunogenicity score > 4, ProtParam instability index < 40, ToxinPred2 non-toxic, and AllerTop non-allergenic, allowing only thirteen of the forty-nine sequences to advance (Table S6). In the second structure-docking round, secondary-structure composition and distribution were first compared (Table 3); full-length models of the thirteen survivors were then generated with AlphaFold-3 and refined through five GalaxyRefine cycles of side-chain repacking and backbone relaxation. The conformation exhibiting the highest GDT-HA together with the lowest MolProbity score was selected for each candidate (Table S7). Subsequent semi-flexible docking against TLR2 and TLR4 was performed with ClusPro 2.0 and ranked by cluster size (docking conformations corresponding to clusters with a large cluster size occur at a high frequency among the large number of randomly generated conformations, which means this protein binding mode is more stable in terms of thermodynamics or spatial structure) and weighted score (a comprehensive energy score calculated by assigning dynamic weights to key energy terms such as van der Waals forces, electrostatic interactions, and solvation free energy, which is used to evaluate the binding stability of complexes) [44]. Priority should be given to models with concentrated conformations within the cluster (i.e., large cluster size) to improve the reliability of the prediction results. On the combined score-sheet EP9158H (N-ESAT6-epitopes-C-HBHA, Figure 5) emerged as the clear winner: its pre- and post-refinement structures are compared in Figure 6A, while Ramachandran plots and ProSA Z-score are supplied in Figure 6B–E.

**Figure 7:**
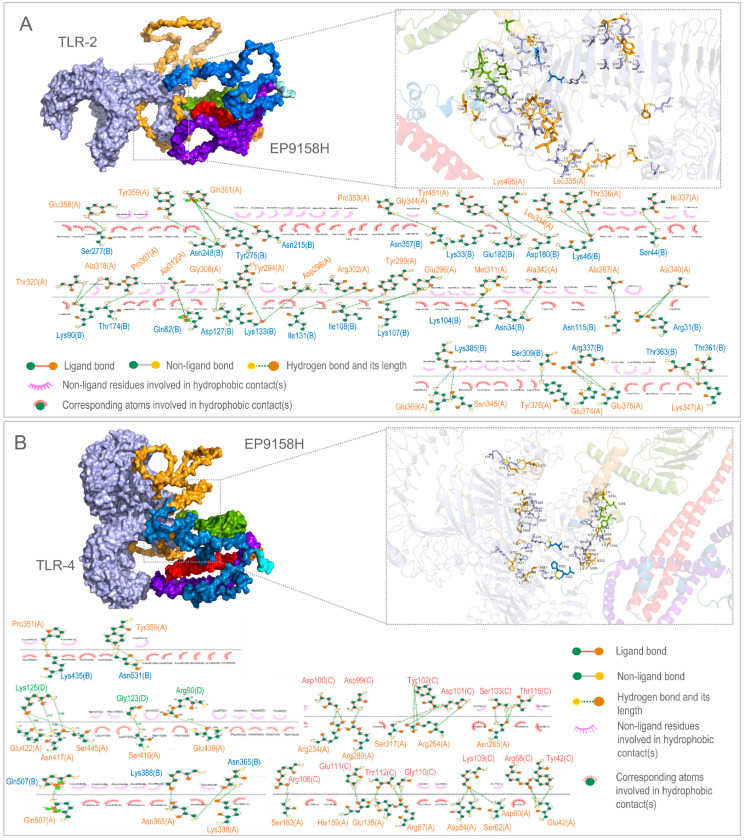
Molecular docking of EP9158H with Toll-like receptors. Predicted binding modes and interaction maps of EP9158H with (**A**) TLR2 and (**B**) TLR4. The 3D surface models (left panels, from ClusPro) show the overall binding interface. The 2D interaction diagrams (right panels, from LigPlot+) detail hydrogen bonds (green dashed lines) and hydrophobic contacts (red arcs). Insets provide magnified views of key interacting residues. The different colors on the spatial structure diagram of the EP9158H vaccine molecule in the picture represent the different components of the vaccine, including linkers, THL epitopes, CTL epitopes, B-cell epitopes, adjuvants, and 6×His.

Detailed docking revealed that EP9158H forms 38 hydrogen bonds with TLR2 at a weighted score of −1359.7, and 36 hydrogen bonds with TLR4 at −1348.3 (Figure 7). The cluster size corresponding to this docking conformation is the largest, outperforming the remaining twelve candidates by a comfortable margin. In parallel, EP9158H delivered a Protein-Sol solubility of 0.531, an IEDB immunogenicity score of 6.738, 95.8% of residues in Ramachandran favored regions, and a ProSA Z-score of −6.18, placing it first in every physicochemical and structural category. Consequently, EP9158H was designated the final vaccine candidate and forwarded to a 100 ns molecular-dynamics simulation and immune-simulation validation.

### 3.5. Structural Stability and TLR-Binding Signature of EP9158H

**Figure 8:**
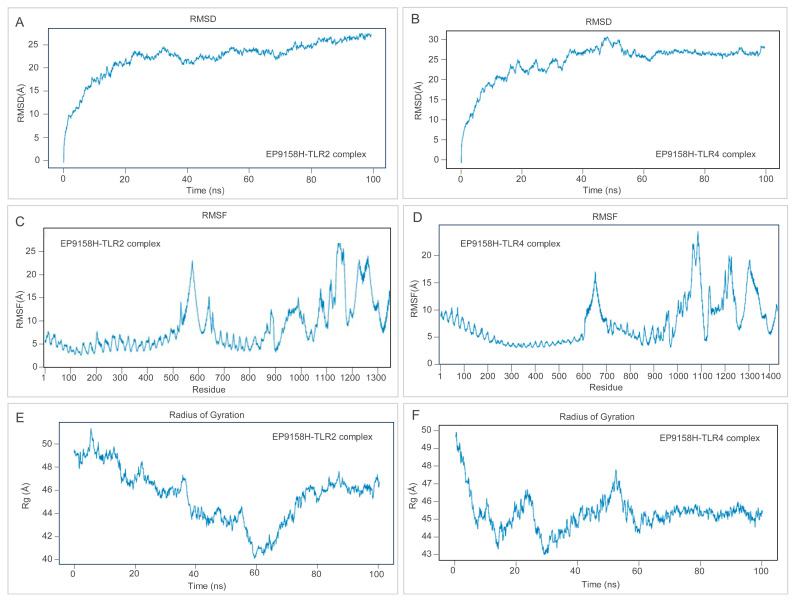
Molecular dynamics (MD) simulation analysis of the vaccine-TLR complexes. (**A**,**B**) Root mean square deviation (RMSD) of the EP9158H-TLR2 and EP9158H-TLR4 complexes over 100 ns, indicating stable binding. (**C**,**D**) Root mean square fluctuation (RMSF) per residue, highlighting flexible regions. (**E**,**F**) Radius of gyration (Rg) plots, reflecting the compactness of the complexes throughout the simulation.

To appraise the durability of the EP9158H–TLR complexes, 100 ns molecular-dynamics trajectories were generated with Desmond (OPLS4, SPC water, 0.15 M NaCl, 300 K, 1 atm), saving snapshots every 100 ps. For EP9158H–TLR2, the Cα RMSD climbed to 22 Å within 20 ns and then fluctuated narrowly between 22 and 26 Å (Figure 8A); the EP9158H–TLR4 counterpart stabilized at 23–27 Å after 15 ns (Figure 8B), each exhibiting a swing of <4 Å and indicating a globally steady architecture. RMSF profiles (Figure 8C,D) showed most residues flexing <10 Å, with peaks around positions 650–700, 1150–1250, and 1275–1300 (RMSF ≈ 20 Å) that correspond to known loop regions. The radius of gyration hovered at 45–46 Å without appreciable expansion or contraction (Figure 8E,F).

**Figure 9:**
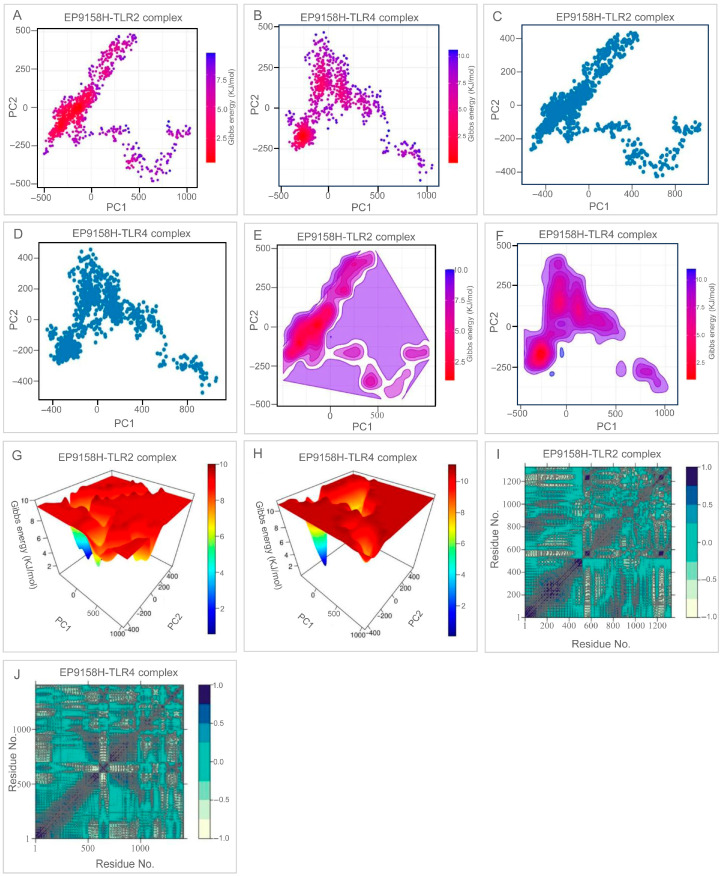
Conformational dynamics and residue correlations from MD trajectories. (**A**,**B**) Principal component analysis (PCA) scatter plots of the first two principal components (PC1, PC2) for the EP9158H-TLR2 and EP9158H-TLR4 complexes, colored by free energy (red: low energy, blue: high energy). (**C**,**D**) Projection of the MD trajectory onto PC1 and PC2 without energy mapping. (**E**,**F**) Two-dimensional free energy landscapes (FELs). (**G**,**H**) Three-dimensional representations of the FELs, showing energy minima corresponding to stable conformational states. (**I**,**J**) Dynamic cross-correlation matrices (DCCM) illustrating correlated (blue) and anti-correlated (red) motions between residue pairs.

Principal-component analysis (Figure 9A–D) revealed that the EP9158H–TLR2 ensemble clusters in the low-value region of PC1–PC2, whereas the TLR4 partner spreads more broadly, reflecting its innate conformational plasticity. Free-energy landscapes (Figure 9E–H) displayed a single deep basin for the TLR2 complex and several shallow minima for the TLR4 assembly, consistent with the receptors’ intrinsic dynamics. Dynamic cross-correlation matrices (Figure 9I,J) showed strongly correlated motions between interface residues of EP9158H and both TLRs, suggesting concerted movements that help preserve binding integrity.

Taken together, the simulations support stable association of EP9158H with TLR2/4 throughout the 100 ns window, with no major dissociation or abnormal conformational drift, thereby providing a structural rationale for downstream immunogenicity assays.

### 3.6. Immune Simulation, Codon Optimization, and mRNA Structure Evaluation

**Figure 10:**
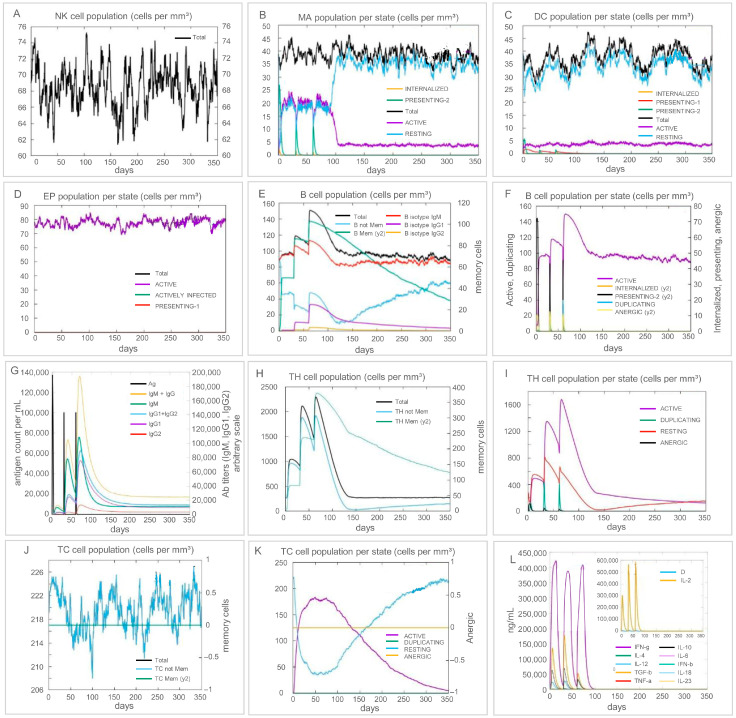
In silico immune simulation profile of EP9158H vaccination. The predicted immune response over time includes: innate immune cell activity (**A**–**D**: NK cells, macrophages, dendritic cells, epithelial cells); B-cell population dynamics and antibody production (**E**–**G**); T-cell responses (**H**–**K**: helper T-cell subsets, cytotoxic T-cell subsets); and cytokine levels (**L**).

ElliPro predicted sixteen discontinuous B-cell epitopes on the surface of EP9158H (3–54 residues, scores 0.694–0.939, Table S8), offering potential targets for antibody responses. C-IMMSIM runs (volume 50, 1050 steps, injections at steps 1-90-180) showed that NK cells peaked around day 100 after priming, macrophages and dendritic cells rose in parallel, and B plus memory B cells expanded continuously; the Th1 and CTL subsets increased, IFN-γ and IL-2 secretion went up, and antibody titers stayed at high levels (Figure 10).

**Figure 11:**
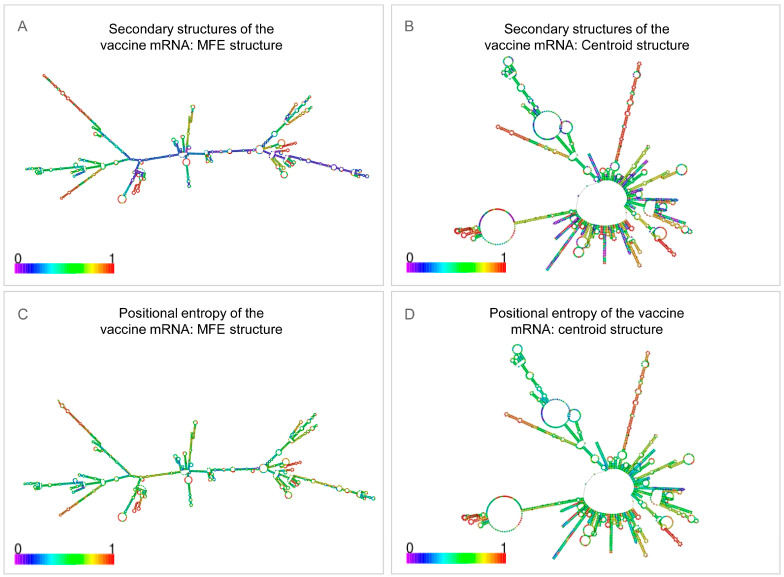
Predicted secondary structure and stability of the EP9158H mRNA sequence. (**A**) Minimum free energy (MFE) secondary structure. (**B**) Centroid secondary structure. (**C**,**D**) positional entropy plots corresponding to the MFE and centroid structures, indicating nucleotide-level flexibility.

**Figure 12:**
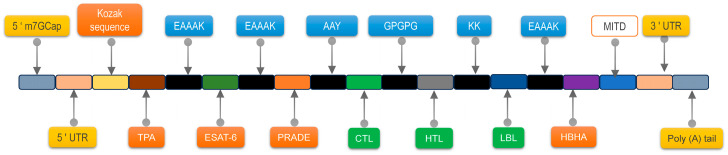
Schematic Diagram of mRNA Structure. From the N-terminus to the C-terminus, the mRNA vaccine is constructed as follows: 5′ m7G Cap–5′ UTR–Kozak Sequence–TPA (Signal Peptide)–EAAAK (Linker)–Psmα4 (Adjuvant)–GPGPG (Linker)–Prade–GPGPG/AAY (Linker)–CTL Epitope–GPGPG–HLT Epitope–KK (Linker)–LBL Epitope–EAAAK–Rpfe (Adjuvant)–6 × His–MITD Sequence–3′ UTR–Poly(A) Tail.

Codon optimization with ExpOptimizer for Homo sapiens yielded a 2418 nt coding sequence, CAI = 0.8, and GC content 61.58%, all within the recommended range. RNAfold forecasts gave a minimum free energy of −1127.5 kcal/mol for the mRNA secondary structure, −740.8 kcal/mol for the centroid structure, −1162.6 kcal/mol for the thermodynamic ensemble, and 0.00% frequency of the MFE structure in the ensemble (Figure 11), indicating satisfactory transcript stability. The mRNA architecture is shown in Figure 12. Collectively, these data support advancing EP9158H to in vivo expression and immunogenicity studies.

## 4. Discussion

The remarkable success of mRNA vaccines against viral infections like COVID-19 and RSV has opened a new era in vaccinology [14,16,17,22,45]. However, applying this powerful platform to bacterial diseases, such as TB, presents greater challenges. Unlike viruses, MTB has a complex life cycle and a large genome [46,47,48,49]. Its antigen expression varies significantly across different stages of infection. This makes it very difficult for a single antigen to provide broad protection [17]. Currently, only two TB mRNA candidates (BNT164a1/b1) have entered phase I trials, underscoring the translational gap that still exists [4,5]. The principal hurdles are the pleomorphic lifestyle of MTB, its thick cell wall that limits antigen release, and the need to orchestrate both CD4^+^ and CD8^+^ immunity against predominantly intracellular epitopes [50]. These challenges have kept most pre-clinical studies confined to single-antigen constructs: nude mRNA-MPT83 reduced lung CFU by 0.6 log_10_ in mice [23], nasal mRNA-Hsp65 achieved a 0.7 log_10_ reduction [24], and a self-amplifying repRNA-ID91 gave 0.85 log_10_ protection only when used as an mRNA-prime/protein-boost regimen [25]. Protection waned after eight weeks and never exceeded that of BCG, highlighting the need for broader epitope coverage and built-in adjuvanticity. Immunoinformatics offers a way forward, but published computational TB mRNA vaccines [51,52,53] stopped at docking or short MD snapshots; none integrated population-wide HLA filtering, dual TLR agonism, and full-length stability testing in a single pipeline.

To address these challenges, we designed a novel mRNA vaccine candidate, EP9158H, using a comprehensive immunoinformatics pipeline. Our approach started with a pool of 13 clinically relevant MTB antigens. Through a multi-step screening process, we constructed a multivalent scaffold (mRV12) containing 60 epitopes (36 CTL, 16 HTL, 8 B-cell). A key innovation was the integration of two TLR agonists—ESAT-6 (TLR2) and HBHA (TLR4)—at opposite ends of the scaffold. To assemble the vaccine, we used a rationally engineered linker set to join the selected epitopes: the flexible linkers AAY and KK, the rigid linker EAAAK, and the GPGPG linker. The latter combines flexibility with local rigidity to mimic natural protein hinge regions, enhancing immune recognition [54]. This dual-agonist design sets EP9158H apart from other computationally designed TB mRNA vaccines [51,52,53], as it aims to synergistically activate innate immunity. Furthermore, compared to our team’s earlier multi-epitope peptide or protein vaccines (e.g., PP13138R, ZL12138L, Table 4) [55,56,57,58,59,60,61], EP9158H represents a platform upgrade. As an mRNA vaccine, it enables endogenous antigen production inside host cells, which is crucial for triggering strong cytotoxic T-cell responses against intracellular pathogens like MTB.

A critical determinant of vaccine efficacy against MTBC diversity is epitope conservation [63]. Our individual BLASTP analysis of all 32 constituent epitopes revealed that 26 (81.25%) demonstrate 100% identity across all MTBC lineages, the six remaining epitopes showed only minor variation (81–100%) involving conservative substitutions at non-anchor positions. The concept of cross-reactivity, even in the face of minor sequence variation, is supported by literature in the field. For T-cell epitopes, sequence identity levels above 85–90% are often considered permissive for cross-reactivity, especially when substitutions are conservative and occur at positions not critical for MHC binding or TCR engagement [64,65,66]. Furthermore, vaccine studies against complex pathogens have demonstrated that epitopes with conservation levels comparable to our lowest (81%) can still contribute to broad protection [67,68,69,70,71,72]. Nonetheless, we acknowledge that our computational predictions require validation, and the functional impact of the observed variations must be conclusively determined through experimental studies, such as heterologous challenge with representative MTBC strains.

A vaccine’s effectiveness depends heavily on the stability of its structure [73,74,75,76]. For a large molecular construct like EP9158H, maintaining a stable shape is essential for proper function. Our molecular dynamics simulations provide reassuring insights. The complexes formed by EP9158H with both TLR2 and TLR4 demonstrated remarkable stability over 100 nanoseconds. Key metrics, like the RMSD, settled into a tight, steady range after an initial equilibration period. Similarly, the radius of gyration (Rg) remained constant, showing no significant unfolding or compaction of the vaccine-receptor complexes [77]. The high binding affinity is explained by the detailed interactions at the interface. EP9158H formed an extensive network of hydrogen bonds with both TLRs (38 with TLR2, 36 with TLR4), resulting in very strong binding energies. This robust and stable interaction suggests that EP9158H can effectively bridge and activate TLR2 and TLR4 signaling, providing a solid structural basis for its predicted adjuvant effect. These stability parameters compare favorably with other published designs [51,52,53].

The great advantage of mRNA vaccines is their ability to produce antigens inside the cell [78,79]. This mimics a natural infection and optimally stimulates the immune system [14,15,18,22,80]. For EP9158H, this means its encoded CTL and HTL epitopes can be efficiently presented to the immune system via two pathways. First, the synthesized protein is processed in the cytoplasm. CTL epitopes are loaded onto MHC-I molecules and presented to CD8^+^ T cells. This activates them to become cytotoxic T lymphocytes (CTLs) [81,82]. These CTLs can then identify and kill host cells infected with MTB by releasing perforin and granzymes [4,5,54]. This is a critical mechanism for eliminating the hidden reservoir of bacteria. Second, some of the vaccine antigen is also processed through the MHC-II pathway, which activates CD4^+^ T cells [83]. Our immune simulations predict a strong Th1-type response, with high levels of IFN-γ and IL-2. This is crucial because IFN-γ is essential for empowering macrophages to kill MTB [84,85,86]. By simultaneously activating both CTLs and helper T cells, EP9158H is designed to mount a coordinated attack against the bacterium. The C-ImmSim results reflect this, showing robust activation of both T-cell types and high antibody titers, outperforming predictions for many other candidate designs [51,52,53].

Despite these encouraging metrics, four limitations must be acknowledged. First, all immunogenicity read-outs are computational in origin; although the algorithms have been validated on viral and cancer datasets, their accuracy for mycobacterial epitopes remains empirically untested [87,88]. Additionally, our vaccine incorporates two TLR agonists. While these adjuvants can markedly enhance immunogenicity, they also carry the theoretical risk of excessive innate activation—potentially culminating in a cytokine storm [89,90,91]. This possibility will be closely monitored and mitigated through rigorous dose-escalation and safety studies in forthcoming animal experiments. Second, while global HLA-II coverage reaches 88.91%, HLA-I coverage drops to 1.40% in Central America and 1.79% in South Africa—gaps that will require region-specific epitope swaps or poly-epitope cassette insertion. Finally, the current work stops at in silico transcript folding and does not address the physicochemical stability of the final LNP formulation, a factor known to influence mucosal trafficking and translational efficiency in the lung [92,93]. Our group will optimize LNP composition and structure by adjusting the phospholipid-to-cholesterol ratio to improve mRNA vaccine encapsulation and delivery efficiency. Additionally, novel delivery carriers—such as solvent-free, continuous manufacturing processes—will be employed to achieve uniform particle size and enhanced stability.

## 5. Conclusions

In conclusion, we have used a rational immunoinformatics approach to design EP9158H, a novel multi-epitope mRNA vaccine candidate for TB. Its innovative design incorporates dual TLR agonists and demonstrates excellent structural stability, strong receptor binding, and promising immunogenic potential in silico. These results provide a strong justification for moving EP9158H into experimental development. Future work will focus on synthesizing the mRNA, testing its immunogenicity and protective efficacy in animal models, and optimizing a delivery system to bring this candidate closer to a much-needed next-generation TB vaccine.

## Figures and Tables

**Table 1:** Prediction of signal peptides, transmembrane domains, and protein localization.

Protein	Signal Peptide Expression			Transmembrane Domain		Localization	
	Signal 6.0	TOP-CONS	Deep-TMHMM	TOP-CONS	Deep-TMHMM	DeepLocPro	TBpred
Rv0287	—	—	—	—	—	Extracellular	Secreted protein
Rv0288	—	—	—	—	—	Extracellular	Secreted protein
Rv1009	+	+	+	—	—	Cytoplasmic Membrane	Cytoplasmic protein
Rv1733c	—	—	—	+	+	Cytoplasmic Membrane	Integral membrane protein
Rv1886c	+	+	+	—	—	Extracellular	Secreted protein
Rv2006	—	—	—	—	—	Cytoplasmic	Cytoplasmic protein
Rv2450c	+	+	+	—	—	Extracellular	Cytoplasmic protein
Rv3131	—	—	—	—	—	Cytoplasmic	Integral membrane protein
Rv3619c	—	—	—	—	—	Extracellular	Secreted protein
Rv3803c	+	+	+	—	—	Extracellular	Secreted protein
Rv3804c	+	—	+	+	—	Extracellular	Secreted protein
Rv3873	—	+	—	—	—	Extracellular	Secreted protein
Rv3876	—	—	—	—	—	Cytoplasmic	Integral membrane protein

**Table 2:** Detailed information on the HTL, CTL, and B-cell epitopes selected to construct the EP9158H vaccine.

Protein	Peptide Sequence	Length	Alleles	Percentile Rank ^a^	Antigenicity Score ^b^	IFN-γ Score ^c^	Immunogenicity Score ^d^	ABC Pred Score ^e^	IC50 ^f^	AllerTOP V 2.0 ^g^	Toxin Pred ^h^	IL-4 ^i^	IL-10 ^j^
**HTL epitopes**													
RpfB	SGMALPVVSAKTVQLNDG	18	HLA-DRB1*09:01	0.43	0.6931	0.32212526				Non	Non	Non	Non
Rv1733c	VSLLTIPFAAAAGTAVQD	18	HLA-DRB1*09:01	0.15	0.7454	0.88663219				Non	Non	Non	Non
Ag85B	LPVEYLQVPSPSMGRD	16	HLA-DRB1*07:01	0.43	0.9243	0.90641281				Non	Non	Non	Non
	QDAYNAAGGHNAV	13	HLA-DQA1*04:01/DQB1*04:02	0.42	1.2979	1				Non	Non	Non	Non
Rv2006	IADVQGGTTQEG	12	HLA-DQA1*05:01/DQB1*03:01	0.03	1.9778	1				Non	Non	Non	Non
RpfE	DAGFDPNLPPPLAPDFLS	18	HLA-DRB3*02:02	0.13	1.2723	0.25969119				Non	Non	Non	Non
Mpt51	VAVAAEPTAKAAPY	14	HLA-DRB1*04:01	0.17	0.5817	0.17280275				Non	Non	Non	Non
Ag85A	GANSPALYLLDGLRAQDD	18	HLA-DRB1*01:01	0.43	0.598	0.92547671				Non	Non	Non	Non
PPE68	QAKTRAMQATAQAAAYT	17	HLA-DQA1*01:02/DQB1*06:02	0.43	0.7682	0.45477448				Non	Non	Non	Non
**CTL epitopes**													
RpfB	LPAPNVAGL	9	HLA-B*07:02	0.06	0.5645		0.08692		42.4	Non	Non		
Rv1733c	ATSAPPRTK	9	HLA-A*11:01	0.01	0.8273		0.03079		36.74	Non	Non		
	FAAAAGTAV	9	HLA-A*68:02	0.29	0.6628		0.17769		18.83	Non	Non		
	QTRHPATATV	10	HLA-A*68:02	0.11	0.5937		0.16889		40.07	Non	Non		
Ag85B	NAAGGHNAV	9	HLA-A*68:02	0.2	1.9957		0.12765		44.56	Non	Non		
Rv2006	ALFVAPVLSL	10	HLA-A*02:03	0.29	0.7261		0.03968		43.17	Non	Non		
	AVVADLAAV	9	HLA-A*02:01	0.26	0.5606		0.11981		19.95	Non	Non		
			HLA-A*02:03	0.13	0.5606		0.11981		5.38	Non	Non		
			HLA-A*02:06	0.07	0.5606		0.11981		4.13	Non	Non		
	DAATLTAAI	9	HLA-A*68:02	0.17	0.7419		0.13338		24.57	Non	Non		
	HTAELDAGV	9	HLA-A*02:06	0.36	1.2021		0.17635		46.79	Non	Non		
			HLA-A*68:02	0.01	1.2021		0.17635		2.92	Non	Non		
RpfE	SINTGNGYY	9	HLA-A*30:02	0.06	0.6527		0.09031		19.99	Non	Non		
Mpt51	ALSFGLGGV	9	HLA-A*02:03	0.11	0.8126		0.13506		4.57	Non	Non		
PPE68	MQATAQAAAY	10	HLA-B*15:01	0.04	0.575		0.06977		4.88	Non	Non		
			HLA-A*30:02	0.35	0.575		0.06977		33.78	Non	Non		
	QATAQAAAY	9	HLA-B*35:01	0.04	0.7038		0.03188		19.69	Non	Non		
	RPGLVAPAPL	10	HLA-B*07:02	0.1	1.0552		0.09424		9.41	Non	Non		
	TEMDYFIRM	9	HLA-B*44:03	0.04	0.5697		0.21448		32.71	Non	Non		
			HLA-B*40:01	0.08	0.5697		0.21448		34.49	Non	Non		
**B cellular epitopes**													
RpfB	KVTERLPLPPNARRVE	16						0.95		Non	Non		
Rv1733c	ATVIDHEGVIDSNTTA	16						0.86		Non	Non		
Ag85B	AGGYKAADMWGPSSDP	16						0.88		Non	Non		
Rv2006	ARGIRLPPGSPTDLTD	16						0.92		Non	Non		
RpfE	AAAGPDAVGFDPNLPP	16						0.97		Non	Non		
Mpt51	NGMWGAPQLGRWKWHD	16						0.95		Non	Non		
Ag85A	DFSGWDINTPAFEWYD	16						0.9		Non	Non		
PPE68	APLAQEREEDDEDDWD	16						0.93		Non	Non		

Abbreviations: HTL, helper T lymphocyte; CTL, cytotoxic T lymphocyte; IFN-γ, interferon-γ; IC_50_, half maximal inhibitory concentration; IL, interleukin. ^a^ The percentile ranking of the selected epitopes; the inclusion criterion was a ranking score <0.5. ^b^ The antigenicity score; epitopes with an antigenicity score >0.5 were selected. ^c^ IFN-γ score; the epitopes with the highest positive scores were selected. ^d^ The immunogenicity score; epitopes were selected in order of score. ^e^ The linear B-cell epitope prediction score; epitopes were selected in order of score. ^f^ The half maximal inhibitory concentration test; epitopes with IC50 value < 50 nM were selected. ^g^ The result of the sensitization test; “Non” indicates that the epitope was non-sensitizing. ^h^ The result of the Toxin; “Non” indicates that the epitope was non-toxic. ^i^ Stimulated cytokine IL-4 secretion. HTL epitopes were selected that do not secrete cytokine IL-4. ^j^ Stimulated cytokine IL-10 secretion. HTL epitopes were selected that do not secrete cytokine IL-10.

**Table 3:** Proportion of secondary structures of vaccines, comparison of tertiary structures before and after optimization, and 3D structural model.

Agonists Contained	Secondary Structure		Tertiary Structure			
	Proportion of Structure Types	Distribution Chart	Region	Before Optimization	After Optimization	3D Structural Model
RpIL	Alpha helix: 37.54%	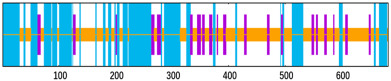	Most favored regions: Additional allowed regions: Generously allowed regions: Disallowed regions:	79.5% 12.8% 2.9% 4.8%	96.5% 2.2% 0.5% 0.7%	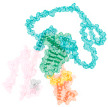
	Extended strand: 12.61%					
	Random coil: 49.85%					
HBHA	Alpha helix: 43.74%	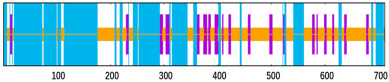	Most favored regions: Additional allowed regions: Generously allowed regions: Disallowed regions:	85.1% 11.2% 1.7% 1.9%	95.5% 4.2% 0.3% 0.0%	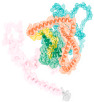
	Extended strand: 11.53%					
	Random coil: 44.73%					
ESAT6 + RpIL	Alpha helix: 51.09%	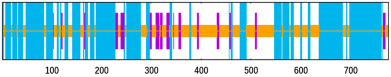	Most favored regions: Additional allowed regions: Generously allowed regions: Disallowed regions:	80.0% 14.3% 2.2% 3.5%	96.7% 2.9% 0.2% 0.3%	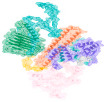
	Extended strand: 8.49%					
	Random coil: 40.41%					
ESAT6 + HBHA	Alpha helix: 53.10%	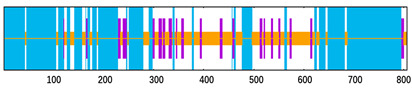	Most favored regions: Additional allowed regions: Generously allowed regions: Disallowed regions:	86.4% 10.3% 1.5% 1.8%	95.8% 3.5% 0.3% 0.5%	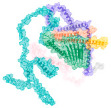
	Extended strand: 10.30%					
	Random coil: 30.60%					
PSMα4 + RpIL	Alpha helix: 40.31%	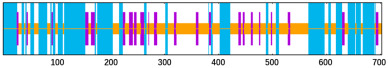	Most favored regions: Additional allowed regions: Generously allowed regions: Disallowed regions:	79.5% 13.1% 3.9% 3.5%	92.9% 5.5% 0.7% 0.9%	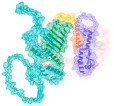
	Extended strand: 12.68%					
	Random coil: 47.01%					
PSMα4 + HBHA	Alpha helix: 48.43%	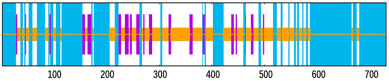	Most favored regions: Additional allowed regions: Generously allowed regions: Disallowed regions:	84.3% 12.7% 1.8% 1.2%	96.6% 3.2% 0.2% 0.0%	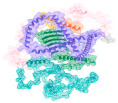
	Extended strand: 10.12%					
	Random coil: 41.45%					
RpIL + ESAT6	Alpha helix: 46.98%	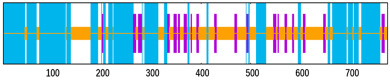	Most favored regions: Additional allowed regions: Generously allowed regions: Disallowed regions:	82.6% 10.9% 3.2% 3.3%	95.6% 3.0% 0.5% 1.0%	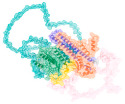
	Extended strand: 10.55%					
	Random coil: 42.47%					
RpIL + PSMα4	Alpha helix: 39.17%	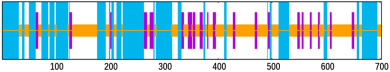	Most favored regions: Additional allowed regions: Generously allowed regions: Disallowed regions:	75.4% 17.7% 3.9% 3.0%	94.5% 3.9% 0.5% 1.1%	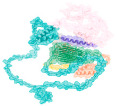
	Extended strand: 12.25%					
	Random coil: 48.58%					
RpIL + Pam2Cys	Alpha helix: 37.70%	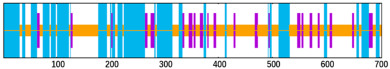	Most favored regions: Additional allowed regions: Generously allowed regions: Disallowed regions:	75.1% 17.0% 3.0% 4.9%	93.3% 4.9% 1.1% 0.7%	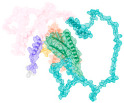
	Extended strand: 13.23%					
	Random coil: 49.08%					
HBHA + ESAT6	Alpha helix: 63.40%	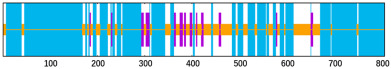	Most favored regions: Additional allowed regions: Generously allowed regions: Disallowed regions:	84.9% 9.5% 2.7% 2.9%	95.6% 3.3% 0.8% 0.3%	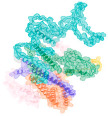
	Extended strand: 6.82%					
	Random coil: 29.78%					
HBHA + PSMα4	Alpha helix: 44.19%	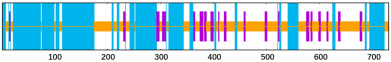	Most favored regions: Additional allowed regions: Generously allowed regions: Disallowed regions:	87.3% 9.9% 1.0% 1.8%	96.3% 2.5% 0.3% 0.8%	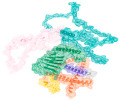
	Extended strand: 11.22%					
	Random coil: 44.60%					
HBHA + Pam2Cys	Alpha helix: 42.62%	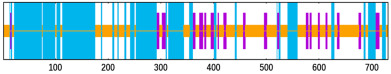	Most favored regions: Additional allowed regions: Generously allowed regions: Disallowed regions:	84.9% 11.9% 2.5% 0.7%	95.0% 4.2% 0.3% 0.5%	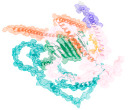
	Extended strand: 11.48%					
	Random coil: 45.90%					
HBHA + PorB	Alpha helix: 45.94%	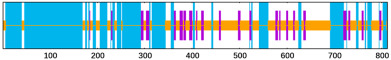	Most favored regions: Additional allowed regions: Generously allowed regions: Disallowed regions:	85.2% 10.6% 2.0% 2.3%	95.0% 3.5% 0.8% 0.8%	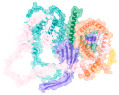
	Extended strand: 11.45%					
	Random coil: 42.61%					

**Table 4:** Comparison of parameters among different vaccines.

Vaccine	Biological Characteristics		Physicochemical Properties		Weighted Score		References
	Immunogenicity	Antigenicity	Solubility	Instability Index	TLR-2	TLR-4	
EP9158H	6.73869	0.7613	0.531	38.44	−1359.7	−1348.3	This study
ZL12138L	4.14449	0.8843	0.47	28.42	−1173.4	−1360.5	[55]
PP13138R	1.44921	0.8968	0.515	28.56	−1167.3	−1255.1	[56]
PP19128R	9.29811	0.8067	0.900675	33.20	−1324.77	−1278	[57]
HP13138PB	2.79	0.87	0.55	33.20	−1224.7	NA	[58]
S7D5L4	1.45499	0.7811	0.462	26.51	NA	−1208.1	[59]
MP3RT	0.61	0.88	0.55	29.65	−1066.4	−1250.4	[62]
CP91110P	4.40091	0.8789	0.485	33.48	−1535.9	−1672.5	[42]

## Data Availability

The original contributions presented in this study are included in the article/Supplementary Materials. Further inquiries can be directed to the corresponding authors.

## Supplementary Materials

The following supporting information can be downloaded at: https://www.mdpi.com/article/10.3390/bioengineering12121378/s1. Table S1: Preliminary screening results of cytotoxic T-lymphocyte (CTL) epitopes. Table S2: Preliminary screening results of helper T-lymphocyte (HTL) epitopes. Table S3: Preliminary screening results of linear B-cell epitopes. Table S4: Physicochemical properties and HLA allele population coverage of the 25 multi-epitope vaccine constructs. Table S5: Physicochemical properties of the 49 vaccine constructs incorporating Toll-like receptor (TLR) agonists. Table S6: Physicochemical properties and molecular docking binding energies of the 13 top-ranking vaccine candidates. Table S7: Comparative scores for the structural models of EP9158H before and after refinement. Table S8: Discontinuous B-cell epitopes in the EP9158H structure predicted by ElliPro.

## Abbreviations

The following abbreviations are used in this manuscript:

BCG	Bacille Calmette–Guérin
CD	Cluster of Differentiation
CFU	Colony-Forming Unit
COVID-19	Coronavirus Disease 2019
CTL	Cytotoxic T lymphocytes
DC	Dendritic Cell
DCCM	Dynamic Cross-Correlation Matrices
EP	Epithelial Cell
FELs	Free-Energy Landscapes
GDT-HA	Global Distance Test–High Accuracy
GRAVY	Grand Average of Hydropathy
HLA	Human Leukocyte Antigen
HTL	Helper T lymphocyte
IFN-γ	Interferon-γ
IL10	Interleukin-10
IL4	Interleukin-4
IVT	In Vitro Transcription
kDa	Kilodalton
MA	Macrophage
MD	Molecular Dynamics
MFE	Minimum Free Energy
MHC	Major Histocompatibility Complex
MTB	*Mycobacterium tuberculosis*
MTBC	*Mycobacterium tuberculosis* complex (MTBC)
MW	Molecular Weight
NK	Natural Killer Cell
NPT	Number of Particles, Pressure, Temperature Constant
PCA	Principal Component Analysis
pI	Isoelectric Point
Rg	Radius of Gyration
RMSD	Root Mean Square Deviation
RMSF	Root Mean Square Fluctuation
RSV	Respiratory Syncytial Virus
SARS-CoV-2	Severe Acute Respiratory Syndrome Coronavirus 2
SPC	Simple Point Charge Model
TB	Tuberculosis
TC	Cytotoxic T Cell
TH	T helper Cell
TLR	Toll-like Receptor
WHO	World Health Organization
